# Microstructuring of Mesoporous Titania Films Loaded with Silver Salts to Enhance the Photocatalytic Degradation of Methyl Blue under Visible Light

**DOI:** 10.3390/nano7100334

**Published:** 2017-10-17

**Authors:** Nicolas Crespo-Monteiro, Anthony Cazier, Francis Vocanson, Yaya Lefkir, Stéphanie Reynaud, Jean-Yves Michalon, Thomas Kämpfe, Nathalie Destouches, Yves Jourlin

**Affiliations:** Univ Lyon, UJM-Saint-Etienne, CNRS, Institut d’Optique Graduate School, Laboratoire Hubert Curien UMR 5516, F-42023 Saint-Etienne, France; anthony.cazier@univ-st-etienne.fr (A.C.); francis.vocanson@univ-st-etienne.fr (F.V.); yaya.llefkir@univ-st-etienne.fr (Y.L.); stephanie.reynaud@univ-st-etienne.fr (S.R.); jean.yves.michalon@univ-st-etienne.fr (J.-Y.M.); thomas.kampfe@univ-st-etienne.fr (T.K.); nathali.destouche@univ-st-etienne.fr (N.D.); yves.jourlin@univ-st-etienne.fr (Y.J.)

**Keywords:** microstructuring, titania, visible, nanoparticles, sol-gel, photocatalysis

## Abstract

The microstructuring of the distribution of silver nanoparticles (NPs) in mesoporous titania films loaded with silver salts, using two-beam interference lithography leading to 1 Dimension (1D) grating, induces variations in the photocatalytic efficiency. The influence of the structuration was tested on the degradation of methyl blue (MB) under ultraviolet (UV) and visible illumination, giving rise to a significant improvement of the photocatalytic efficiency. The periodic distribution of the NPs was characterized by transmission electron microscopy (TEM), high-angle annular dark field scanning transmission electron microscopy (HAADF-STEM) and scanning electron microscopy (SEM).

## 1. Introduction

Titanium dioxide (TiO_2_) is one of the most investigated and widely used photocatalyzers for the photodegradation of pollutants in water and air [1]. Nevertheless, as a result of its large band gap, the photocatalytic activity of TiO_2_ is only activated under ultraviolet (UV) light, which limits its use for indoor-environment applications [2]. Therefore, the development of photocatalysts showing a high activity under visible light irradiation is needed in order to be able to use the sunlight or rays from artificial sources more effectively in photocatalytic reactions. Many studies have attempted to develop visible light-sensitive photocatalysts using, for example, TiO_2_ doped with metallic nanoparticles (NPs) such as Au, Ag or Cu [2,3,4,5]. This approach seems to be of interest for improving the photocatalytic effect in visible light due to the localized surface plasmon resonance band (LSPR) of the metallic NPs, which induces a high absorption in the visible range. At the interface between metallic NPs and TiO_2_, there is a potential barrier (Schottky barrier) that is low enough to allow the excitation of electrons at the surface of the metallic NPs in the conduction band of TiO_2_. The released charges lead to the same photocatalytic effect as in TiO_2_ excited by UV. However, any change in the size and/or shape of the metallic NPs and their distribution and concentration, as well as the optical properties of their environment, will have significant effects on their LSPR and consequently on the overall efficiency of the device [5,6,7]. This can even lead to a counterproductive effect; the non-resonant metallic NPs can act as recombination sites for the photo-generated electrons [5]. Despite extensive investigations, most of the developed systems are not suitable for practical indoor applications because of both the small amount of light present in this environment and the low light-harvesting efficiency of the devices [2,8]. 

In this paper, we propose to increase the photocatalytic efficiency of TiO_2_ films loaded with silver salts, using a direct film microstructuration and periodic NP distribution close to the films’ surface using UV laser interference lithography. The structures are characterized by transmission electron microscopy (TEM), high-angle annular dark field scanning transmission electron microscopy (HAADF-STEM) and scanning electron microscopy (SEM). Their photocatalytic efficiency has been obtained from the degradation of methyl blue (MB) during UV and visible illumination. 

## 2. Results

**Figure 1:**
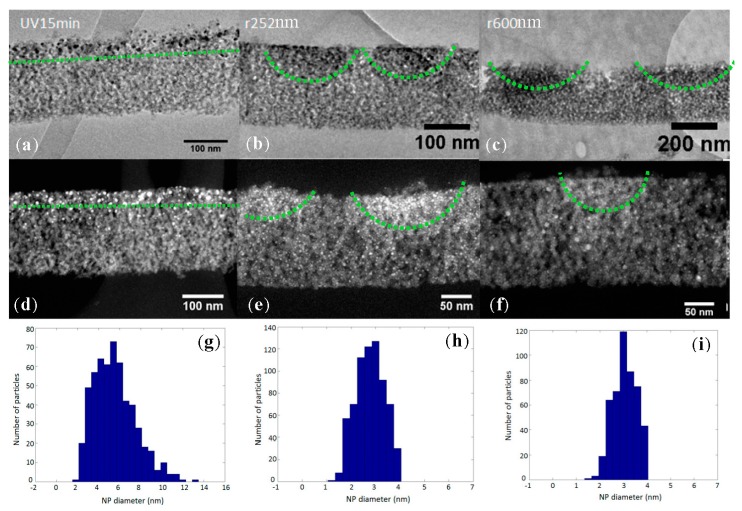
Transmission electron microscopy (TEM) (**a–c**) and high-angle annular dark field scanning transmission electron microscopy (HAADF-STEM) (**d**–**f**) images of cross-section of samples UV15min (**a**,**d**), r252nm (**b**,**c**) and r600nm (**e**,**f**). Nanoparticle (NP)-size histograms deduced from image processing carried out on the HAADF-STEM images of samples UV15min (**g**), r252nm (**h**) and r600nm (**i**).

**Figure 2:**
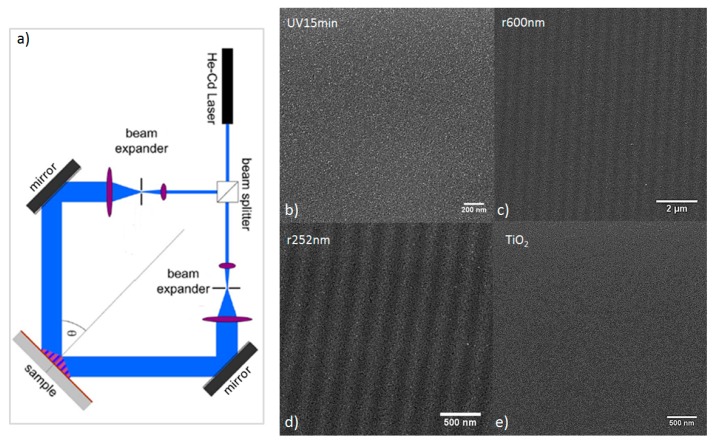
(**a**) Sketch of the holographic bench. Scanning electron microscopy (SEM) images of samples UV15min (**b**), r600nm (**c**), r252nm (**d**) and TiO_2_ (**e**).

**Figure 3:**
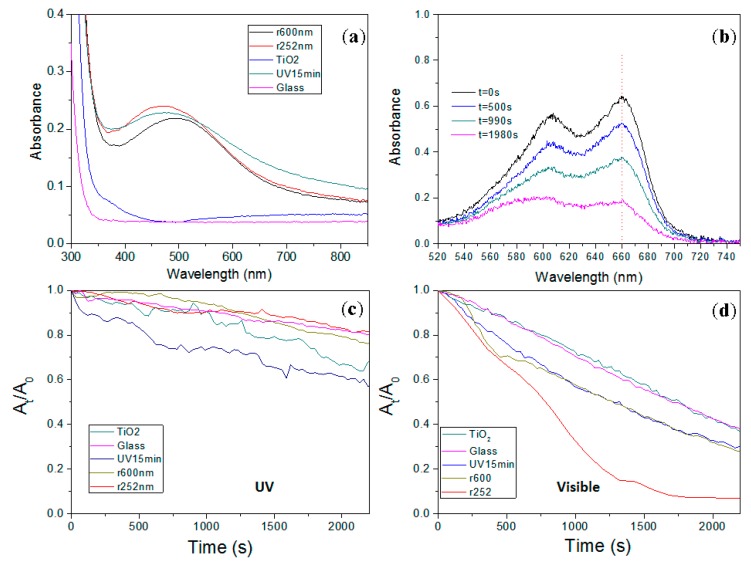
(**a**) Absorbance spectra of glass substrate (glass), TiO_2_, UV15min, r252nm and r600nm samples. (**b**) Absorbance variation of methyl blue (MB) solution during the visible illumination of the sample r600nm. (**c**,**d**) Absorbance variation at 660 nm normalized by the absorbance at the initial state (*t* = 0 s) for the different samples under ultraviolet (UV) or visible illumination, respectively.

The films used in this work were mesoporous TiO_2_ xerogel films deposited on glass substrates. After deposition, the films were doped with silver salts by soaking them for 1 h in an aqueous ammoniacal silver nitrate solution at 0.75 M. After synthesizing the films, different treatments were carried out on their surface. The first was a homogeneous UV illumination for 30 min with two lamps emitting at 254 nm and each delivering a power of 15 W. This illumination allowed the silver salts to be reduced and a homogenous distribution of silver NPs in the uppermost 80–100 nm layer of the film to be produced (Figure 1a,d and Figure 2b). Silver NP growth inside the mesoporous TiO_2_ matrix [9] leads to a certain diameter distribution of the silver NPs, which has been estimated by image processing [10] to be in the range of 1 to 12 nm in diameter with a maximum particle size distribution of around 5 nm in diameter (Figure 1g). Before illumination, the films were transparent and the spectroscopic measurement (PerkinElmer Lambda 900) showed no absorption bands in the UV or visible range, but after illumination, a large absorption band centered around 460 nm appeared (Figure 3a). This absorption band was due to the LSPR resulting from the presence of silver NPs in the TiO_2_ matrix. 

In order to test the influence of microstructuring on the film of silver NPs, two different gratings were realized on the film’s surface, corresponding to a 1 Dimension (1D) periodic index variation close to the film’s surface. The design of the grating was optimized to couple the desired incident wavelength into the film. The first grating coupled the light at a wavelength inside the LSPR (~430 nm), whilst the second coupled the light at a wavelength outside the LSPR (~1020 nm). The gratings were fabricated by two-beam interference lithography (Figure 2a), using a laser of 325 nm wavelength delivering a continuous wave power of 100 mW. The polarized laser beam was split into two arms, which were recombined on the sample, where the overlap created an interferogram during the illumination in the form of a 1D periodic intensity modulation, whose fringe period (Λ) was fixed by the angle (θ) and the wavelength (λ) according to the equation Λ=λ/(2·sin(θ)). The mirrors were mounted on rotation stages and the substrate holder was placed on a rail to allow for automatic tuning of the period. The exposure time for each structure was very long at about 1.5 h, and vibrations had to be kept to a minimum by mounting the optical bench on an air cushion table to filter low-frequency vibrations. Additionally, one of the interferometer arms used a mirror mounted on a piezoelectric motor controlled in a feedback loop by a measurement of the interference fringe positions allowing for a stabilization of the fringe pattern against air convection effects and higher-frequency vibrations. Considering an average refractive index of 1.7 for mesoporous TiO_2_ films (index obtained by ellipsometry measurement), the fringe period required to obtain a grating of NPs capable of coupling the light at the wavelengths of 430 and 1020 nm into the TiO_2_ matrix were 430/1.7 = 252 nm (sample r252nm) and 1020/1.7 = 600 nm (sample r600nm), respectively.

Figure 1 shows images of the realized gratings, obtained by TEM (Figure 1b,c) and HAADF-STEM (Figure 1e,f), and Figure 2 shows the images of gratings by SEM (Figure 2c,d). For the two structures, most of the silver NPs were localized in the first 80–100 nm from the film’s surface and had a diameter of around 2–4 nm with a maximum at 3 nm (Figure 1h,i). The period obtained for the sample r252nm was ~252 nm and this was ~600 nm for the sample r600nm. The NPs induced film coloration and an absorption band in the visible range. The absorbance spectra recorded in the visible range (Figure 3a) showed a similar level of absorbance for all samples with NPs (r252nm, r600nm and UV15min). For these different samples, the degradation of MB was studied over 40 min under UV light by monitoring the absorbance of the MB solution at a wavelength of 660 nm (Figure 3c). After examining the behavior for UV illumination, the photocatalytic activity of the fabricated films was also tested under visible light exposure by investigating the MB degradation using a solar simulator lamp with a cut-off filter at 400 nm and monitoring the absorbance of the MB solution (Figure 3b). Figure 3d shows the absorbance variation at a wavelength of 660 nm during the 40 min of illumination.

## 3. Discussion

The results obtained during UV illumination (Figure 3c) show that the samples with NP gratings had a degradation rate similar to a non-photocatalytic sample (glass substrate), which means that the samples were essentially not photocatalytic under UV light. The mesoporous TiO_2_ film, which had a size of pores between 5 and 15 nm (Figure 2e), had a slightly higher degradation rate. The low degradation efficiency of the TiO_2_ film could be explained by the fact that it was mainly amorphous [11] and therefore not very catalytic under UV light. A better result was obtained for the sample with a homogeneous distribution of silver (UV15min sample). It is well known that the quantity of silver NPs present inside the TiO_2_ matrix can improve [12] (by limiting the recombination of electron–hole pairs photogenerated in the TiO_2_) or inhibit [13] (NPs serving as a recombination site for photo-induced charges) the photocatalytic effect of TiO_2_. If we assume that the presence of silver NPs improves the photocatalytic effect of the TiO_2_ matrix, the samples with silver NPs (r252nm and r600nm films) should have been more photocatalytic than samples without them (TiO_2_). However, the yield was lower than for the pure TiO_2_ films. Consequently, silver NPs seem to inhibit the photocatalytic effect of TiO_2_ rather than improve it. The improvement of the photocatalytic effect could also be due to the increase in crystallization of the TiO_2_ matrix (TiO_2_ crystallized in anatase phase is more photocatalytic than amorphous TiO_2_). Initially, the used films are amorphous, but during UV or visible illumination, even if a low intensity is used, the growth or oxidation of silver NPs can induce a temperature rise that can initiate in their vicinity the crystallization of TiO_2_ in nanocrystals of anatase, brookite or rutile phase [11]. The increase in the number of silver NPs can thus lead to a greater number of anatase TiO_2_ nanocrystals in the film and consequently improves its photocatalytic efficiency. Currently, the influence of each mechanism is unclear and remains to be studied.

During the visible illumination, the sample with a NP grating period of 252 nm showed a better photocatalytic activity (Figure 3d); it allowed for an increasing of the degradation rate of MB by a factor of 2.5 compared to the pure TiO_2_ sample, and by a factor of 1.75 compared to the UV/TiO_2_ sample. The sample with a NP grating period of 600 nm showed a MB degradation similar to that of the sample with the homogeneous distribution of silver NPs. The degradation rates obtained were not only due to the films but also to the light irradiation conditions. If a sample with no photocatalytic activity (glass substrate) was exposed to the same illumination conditions (Figure 3d), one could also observe a degradation of MB, but in this case the degradation time was shorter (the same overall absorbance variation of MB was obtained 7 min later). If a mesoporous TiO_2_ film without silver salts (Figure 3d) was exposed to the same illumination condition, the degradation rate of MB was the same as for a glass substrate; that is to say that the mesoporous TiO_2_ films had no photocatalytic activity under visible irradiation. This confirmed, as it has already been shown in several articles, that the presence of silver NPs in the TiO_2_ matrix is essential to its photocatalytic activity in the visible range [5]. Furthermore, these results show that the distribution of the silver NPs in the TiO_2_ matrix also has an impact on the degradation of MB under visible light. A suitable structuring of the distribution of the silver NPs in the form of a periodic grating allows for increasing (sample r252nm) the photocatalytic efficiency of the TiO_2_/Ag films; however, the parameters need to be chosen carefully, as it has been shown that it is also possible to decrease (sample r600nm) the photocatalytic efficiency. For the sample r252nm, the periodic structuring allowed for an increase in the amount of light absorbed by the films at wavelengths comprised in the LSPR band, increasing the number of available charges. However, the structuring could also change the photocatalytic efficiency by influencing the electron–hole recombination, which is an important point that needs to be clarified in subsequent studies.

## 4. Materials and Methods

The process to synthetize mesoporous TiO_2_ films is detailed in [14]. Their thicknesses were estimated by profilometry (Dektak XT, Bruker, Wissembourg, France) and ellipsometry (SEMILAB GES5-E, Semilab, Budapest, Hungary) to be 150 ± 50 nm, and the porosity volume fraction was estimated at 27%. The size of their pores varied between 5 and 15 nm. Initially the films were mostly amorphous and transparent [11].

The photocatalytic reaction system for UV illumination was composed of two UV lamps of 15 W emitting at 254 nm. For illumination in the visible spectrum, the system consisted of a solar simulator lamp (Newport 94011A Sol series Solar Simulator, Newport, Irvine, CA, USA) equipped with a cut-off filter at 400 nm. A drop of 500 µL of MB (Aldrich, Saint Louis, MO, USA) at 10^−4^ M was deposited on the films’ surface, using substrates of size 2.5 × 2.5 cm^2^. After 30 min in the dark, at room temperature in air, the films were exposed in the UV and in the visible spectrum. During the exposition, the absorbance of the MB solution was recorded every 2 min by a UV–visible spectrometer (Ocean Optics HR2000+, Ocean Optics, Winter Park, FL, USA). MB degradation was detected by measuring the absorption at a wavelength of 660 nm. For UV illumination, a white lamp with an intensity of 1 µW·cm^−2^ was added to allow for an absorbance measurement at 660 nm. All experiments were conducted at room temperature in air.

## 5. Conclusions

In summary, we have shown that microstructuring of a TiO_2_/Ag film influences the photocatalytic efficiency of films on the degradation of MB under visible light. A film for which silver NPs are distributed in the form of a periodic 1D grating fabricated by two-beam interference lithography, whose period has been chosen to couple more light in the film at the wavelength of the localized surface plasmon resonance of the silver NPs allows for an increasing of the photocatalytic efficiency of the TiO_2_ films in white light by a factor of 2.5 compared to a film without NPs and by a factor of 1.75 with respect to a film with a homogeneous distribution of the NPs. For the opposite, a film with an unsuitable structuring (wavelength coupled outside the LSPR of silver NPs) decreases the photocatalytic efficiency of the device relative to a film with a homogeneous distribution. We also showed that under UV illumination, the structuring did not have a measurable effect, and the photocatalytic behavior on the degradation of the MB was not very effective overall. The increase in the photocatalytic efficiency shown in this work can make it possible to consider microstructuring of TiO_2_/Ag films for applications in the field of indoor air treatment, which is currently limited by the low efficiency of the homogeneous TiO_2_/Ag films, in particular as a result of light sources present in this environment, whose intensities are comparably low with a weak amount of UV light. Instead of using a linear grating, the TiO_2_/Ag film can be microstructured with a two-dimensional grating of NPs and/or a two-dimensional topographic grating, which will allow a further increase in the photocatalytic efficiency in the visible range by taking into account the (usually) non-polarized nature of natural light.
